# RNA silencing of integrin-linked kinase increases the sensitivity of the A549 lung cancer cell line to cisplatin and promotes its apoptosis

**DOI:** 10.3892/mmr.2015.3471

**Published:** 2015-03-11

**Authors:** XIAOZHEN ZHAO, ZHENYE XU, ZHONGQI WANG, ZHONGHUA WU, YABIN GONG, LIJUAN ZHOU, YI XIANG

**Affiliations:** 1 Department of Oncology, Longhua Hospital, Shanghai University of Traditional Chinese Medicine, Shanghai 200032, P.R. China; 2 Experiment Center for Science and Technology, Shanghai University of Traditional Chinese Medicine, Shanghai 201203, P.R. China

**Keywords:** lentivirus, RNA interference, integrin-linked kinase, cisplatin, lung cancer

## Abstract

The expression of integrin-linked kinase (ILK) has been reported to be involved in the regulation of integrin-mediated processes, including cancer cell proliferation, migration and invasion. Previous studies have demonstrated that inhibition of ILK may be an underlying approach for treating cancer. However, whether the knock down of ILK affects growth and apoptosis of lung cancer cells remains to be elucidated. Importantly, whether downregulation of ILK increases the sensitivity of lung cancer cells to cisplatin and amplifies cell apoptosis also remains to be elucidated. In the present study, ILK downregulation was mediated by lentivirus-mediated RNA interference. The expression levels of associated genes were determined by reverse-transcription quantitative polymerase chain reaction and western blotting. Cell proliferation was evaluated using a modified 3-(4,5-dimethylthiazol-2-yl)-2,5-diphenyltetrazolium bromide assay and clone formation assay. The cell cycle and apoptosis were analyzed using flow cytometry. The current data revealed that lentivirus-mediated ILK gene silencing alone inhibited A549 cell proliferation and promotes cell cycle arrest, however, had no detectable effect on cell apoptosis. However, combined treatment with lentivirus-mediated ILK interference and cisplatin chemotherapy induced significantly more cell apoptosis than mono-chemotherapy or knockdown. The increased cell apoptosis and proliferation inhibition were attributed to abnormal downstream protein expression of ILK, including phospho-glycogen synthase kinase 3β, p-AKT, activator protein-1, β-catenin, cyclin D1 and matrix metalloproteinase-9. ILK inhibition may suppress the proliferation of A549 and increase A549 sensitivity to cisplatin. The combined treatment of ILK gene knockdown and chemotherapy has the potential to improve anticancer efficacy.

## Introduction

Lung cancer is currently the leading cause of cancer-associated mortality worldwide, being responsible for an estimated 87,750 mortalities in males and 72,590 in females in 2012 (1). The majority of patients have advanced disease (stage III/IV) at initial diagnosis, which contributes to the poor prognosis of these patients. It is reported that the 5-year overall survival rate is <5% among patients with advanced-stage disease (2). Chemotherapy, including cisplatin (CDDP), carboplatin and oxaliplatin, remains a crucial treatment for advanced patients (3). Platinum drugs cross-link at the nucleophilic centers on the DNA of tumor cells, forming intra- (on the same strand) and/or inter-strand (between two opposite strands) adducts between guanines or guanine and adenine, which inhibit DNA replication and trigger cell cycle arrest and apoptosis (4,5). However, the major limitation in the clinical applications of platinum compounds is the development of tumor drug resistance (6–8). Therefore, it is essential to develop more effective treatment strategies to overcome this drug resistance.

Integrin-linked kinase (ILK), a highly conserved, 59 kDa serine/threonine kinase, has been implicated in the regulation of various biological processes that are crucial to the progression of malignant disease (9). ILK expression and activity have been revealed to be increased in association with tumor grade, T status, lymph node metastasis and survival in lung cancer patients (10–12). ILK promotes lung cancer cell migration and invasion via upregulation of matrix metal-loproteinase-9 (MMP-9) (13) and epithelial-mesenchymal transition-associated genes, including vimentin, fibronectin, Snail and Slug (14). Further studies indicate that silencing ILK through targeting small interfering RNA (siRNA) inhibits cell proliferation and growth, induces cell cycle arrest and the apoptosis of bladder (15) and pancreatic cancer cells (16), suggesting the inhibition of ILK may be a novel approach for treating lung cancer. Notably, Song *et al* (17) previously demonstrated that downregulation of ILK by siRNA arrests the growth and increases the CDDP sensitivity and apoptotic rate of human gastric cell line cells that are resistant to SGC7901/CDDP. Thus, it is hypothesized that there may be a synergistic interaction between downregulation of ILK and CDDP administration for treating lung cancer by creating cytotoxic DNA lesions and affecting apoptosis in lung cancer A549 cells. To the best of our knowledge, the present study is the first to examine this mechanism.

## Materials and methods

### Cell culture

The human lung adenocarcinoma cell line A549 and human embryo kidney (HEK) 293T cells (American Type Culture Collection, Manassas, VA, USA) were maintained in Dulbecco’s modified Eagle’s medium (Invitrogen Life Technologies, Carlsbad, CA, USA) containing 10% fetal bovine serum (Invitrogen Life Technologies) and cultured in a humidified atmosphere of 5% CO_2_ at 37°C.

### Construction of lentiviral vectors expressing siRNA targeting ILK and transfection

The oligonucleotides encoding a negative control (NC) siRNA with no homology to the human genome (5′-AAT GTA CTG CGC GTG GAG A-3′) and ILK siRNA (5′-CCT TCA ACT TTG TGC TCA T-3′) were designed and synthesized by Shanghai Jikai Gene Chemical Co., Ltd, (Shanghai, China) and cloned into the *Age* I/*Eco*RI linearized pGCSIL-GFP viral vector (GeneChem, Shanghai, China) to generate the lentiviral vectors. Expression of the lentiviral shRNA was confirmed by DNA sequencing. Plasmids along with 20 *μ*g of pGCSIL-shILK or -shNC lentiviral vectors, 15 *μ*g of packaging vectors pHelper 1.0 and 10 *μ*g of packaging vectors pHelper 2.0 were mixed with 200 *μ*l of Opti-MEM and 15 *μ*l of Lipofectamine 2000 and then transfected into HEK293T cells. After 48 h transfection, lentiviruses were harvested in serum-free medium, filtered and concentrated in primed Centricon Plus-20 filter devices (Millipore, Billerica, MA, USA). Subsequently, A549 cells were infected with ILK-shRNA lentivirus or control lentivirus at a multiplicity of infection of 20. The number of green fluorescent protein (GFP)-positive cells was determined microscopically (MicroPublisher 3.3RTV; Olympus, Tokyo, Japan) three days post-transduction.

### RNA extraction and reverse transcription quantitative polymerase chain reaction (RT-qPCR)

Total RNA was extracted from cells with TRIzol reagent (Invitrogen Life Technologies, Carlsbad, CA, USA) according to the manufacturer’s instructions. Gene expression was detected by RT-qPCR using the standard SYBR Green RT-PCR kit (Takara Bio, Inc., Shiga, Japan). Briefly, the cDNA was synthesized using the RevertAid First-Strand cDNA Synthesis kit (Fermentas, Vilnius, Lithuania) according to the manufacturer’s instructions. The specific primer pairs and the amplified products were as follows: *ILK* (212 bp), sense 5′-TCCACCTGCTCCTCATCC-3′ and anti-sense 5′-CCTCATCAATCATTACACTACGG-3′ and *GAPDH* (121 bp), sense 5′-TGACTTCAACAGCGACACCCA-3′ and antisense 5′-CACCCTGTTGCTGTAGCCAAA-3′. The relative levels of gene mRNA transcripts were normalized to the internal control *GAPDH*. Relative gene expression was quantified using the GraphPad Prism 4.0 software (GraphPad Software, San Diego, CA, USA).

### Western blotting

Cells were lysed in 0.1 ml lysis buffer (0.1% SDS, 1% NP-40, 50 mM HEPES, pH 7.4, 2 mM EDTA, 100 mM NaCl, 5 mM sodium orthovanadate and 1% protease inhibitor mixture set I; Calbiochem, San Diego, CA, USA) on ice for 30 min. Following centrifugation at 13,400 × g (Eppendorf centrifuge 5415 D; Eppendorf AG, Hamburg, Germany) for 15 min, the supernatants were removed and total protein concentration was determined using a bicinchoninic acid protein assay kit (Pierce Biotechnology, Inc., Rockford, IL, USA). Proteins (20 *μ*g) were separated in 10% sodium dodecyl sulfate polyacrylamide gel electrophoresis and transferred onto a polyvinylidene difluoride (PVDF) membrane at 400 mA for 2 h. Following blocking at room temperature in 5% bovine serum albumin for 1.5 h, PVDF membranes were incubated with the following antibodies: Rabbit anti-ILK polyclonal antibody (Santa Cruz Biotechnology, Inc., Santa Cruz, CA, USA), rabbit anti-phospho-glycogen synthase kinase (GSK)-3β-S9 monoclonal antibody (1:1,000), rabbit anti-p-Akt-S473 poly-clonal antibody (1:1,000), rabbit anti-MMP-9 (1:200), rabbit anti-activator protein (AP-1; 1:800), rabbit anti-β-catenin polyclonal antibody (1:1,000), rabbit anti-cyclin D1 monoclonal antibody (1:500), rabbit anti-vascular endothelial growth factor polyclonal antibody (VEGF; 1:500), all purchased from Cell Signaling Technology, Inc. (Danvers, MA, USA) and mouse anti-GAPDH monoclonal antibody (Santa Cruz Biotechnology, Inc; 1:4,000), followed by incubation with the correspondent peroxidase-conjugated secondary antibodies (goat anti-rabbit lgG; 1:4,000; goat anti-mouse lgG; Santa Cruz Biotechnology, Inc.; 1:4,000). Chemiluminescent detection was performed with the enhanced chemiluminescence kit (Pierce Biotechnology, Inc.).

### 3-(4,5-dimethylthiazol-2-yl)-2,5-diphenyltetrazolium bromide (MTT) assay for assessment of proliferation and drug sensitivity

The experimental cells in the exponential phase of growth were plated at a final concentration of 2×10^3^ cells/well in 96-well culture plates for different culture time periods. Varied concentrations of CDDP (5, 10, 15, 20 and 25%) were added to each well and the cells were incubated for 72 h. MTT (10 *μ*l, 5 mg/ml) was then added. Following an additional 4 h of incubation, the reaction was terminated by removal of the supernatant and addition of 100 *μ*l dimethyl sulfoxide for 30 min. The optical density of each well was measured at 490 nm using an ELISA reader (ELx808; Bio-Tek Instruments, Winooski, VT, USA).

### Detection of apoptosis by flow cytometry

Cells were stained with fluorescein isothiocyanate (FITC)-labeled annexin-V and simultaneously with propidium iodide (PI), to discriminate intact cells (annexin-/PI-) from apoptotic cells (annexin+/PI-) and necrotic cells (annexin+/PI+). A total of 1.0×10^6^ cells were washed twice with ice-cold phosphate-buffered saline (PBS) and incubated for 30 min in a binding buffer (1 *μ*g/ml PI and 1 *μ*g/ml FITC-labeled annexin-V), respectively. Fluorescence-activated cell sorting analysis for annexin-V and PI staining was performed using a flow cytometer (Beckman Coulter, Inc., Fullerton, CA, USA). All experiments were performed in triplicate.

### Cell cycle assay

Cells were seeded into a 6-well plate, harvested by centrifugation at 13,40 × g for 5 min (Eppendorf centrifuge 5415 D; Eppendorf AG). Following being washed twice in pre-cooled PBS (pH 7.4), cells were fixed in 70% alcohol. The percentage of cells in each stage of the cell cycle was determined by staining with PI. The analysis of cell cycle distribution was performed by FACScan flow cytometer (Becton-Dickinson, Franklin Lakes, NJ, USA) in accordance with the manufacturer’s instructions.

### Colony-forming assay

Exponentially growing cells transfected with ILK-RNAi-lentivirus and negative control lentivirus were suspended in complete growth medium and seeded in 6-well plates at 200 cells per well. The plates were maintained at 37°C in a humidified incubator with 5% CO_2_ for 2 weeks. The visible colonies were subsequently recorded under an inverted fluorescence microscope (MicroPublisher 3.3RTV; Olympus). Following fixation in paraformaldehyde, the colonies were subjected to Giemsa (Karyomax; Gibco, Grand Island, NY, USA) staining for 10 min followed by acquisition of images with an Olympus C5050 digital camera attached to an Olympus CKX1 inverted microscope (Olympus).

### Statistical analysis

Data are expressed as the mean ± standard deviation. Statistical analysis was performed using SPSS 11.0 software (SPSS, Inc., Chicago, IL, USA). The difference between two groups was analyzed using Student’s t-test. P<0.05 was considered to indicate a statistically significant difference.

## Results

### ILK expression in A549 cells following treatment with lentivirus-mediated RNAi

**Figure 1:**
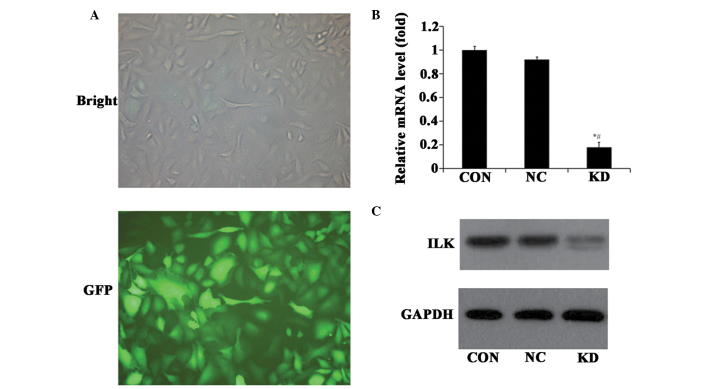
Downregulation of ILK expression by lentivirus-mediated RNAi. (A) Expression of lentivirus-mediated RNAi in A549 cells. At 3 days after infection with lentivirus, A549 cells were visualized using a fluorescence microscope. The phase contrast (bright) and fluorescence (GFP; green) images were captured with the same exposure times at magnification, ×200. (B) Relative levels of *ILK* mRNA transcripts were analyzed by RT-qPCR. Data are presented as the mean ± standard deviation of a representative experiment performed in triplicate (n=3; ^*^P<0.05, compared with the Con group; ^#^P<0.05, compared with the NC group). (C) Silencing effects of ILK protein were measured by western blot analysis. CON, parent cells; NC, cells transfected with negative control RNAi; KD, cells transfected with ILK specific RNAi; ILK, integrin-linked kinase; GFP, green fluorescent protein.

At 3 days after transfection, A549 cells were visualized using a fluorescence microscope and the mRNA and protein levels of ILK were also analyzed. The results revealed that >80% of cells had green fluorescent signals compared with the bright field (Fig. 1A). The level of ILK mRNA in ILK specific-RNAi infected cells was significantly decreased by ~70% (P<0.05), compared with the control RNAi-infected cells (Fig. 1B). In accordance with the silencing of mRNA expression, ILK protein was also down-regulated in ILK RNAi cells (Fig. 1C). These findings suggest that ILK-specific RNAi may downregulate ILK expression efficiently.

### Effect of ILK knockdown on cell proliferation, clone forma- tion, the cell cycle and cell apoptosis in vitro

**Figure 2:**
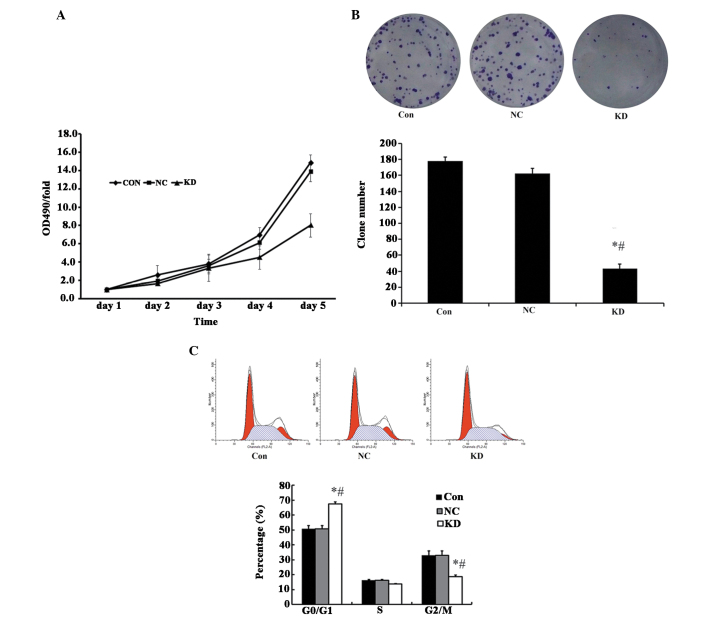
Functional effect of ILK knockdown. (A) Knockdown of ILK inhibited the cell proliferation of A549 cells [measured using a 3-(4,5-dimethyl-thiazol-2-yl)-2,5-diphenyltetrazolium bromide assay], (B) reduced the colony formation ability (measured by colony formation assay), (C) augmented the proportion of G0/G1 phase (the proportion of different cell cycle phases was quantitated by propidium iodide staining followed by flow cytometric analysis). ^*^P<0.05, compared with the Con group; ^#^P<0.05, compared with the NC group. CON, parent cells; NC, cells transfected with negative control RNAi; KD, cells transfected with ILK specific RNAi; ILK, integrin-linked kinase; OD, optical density.

**Figure 3:**
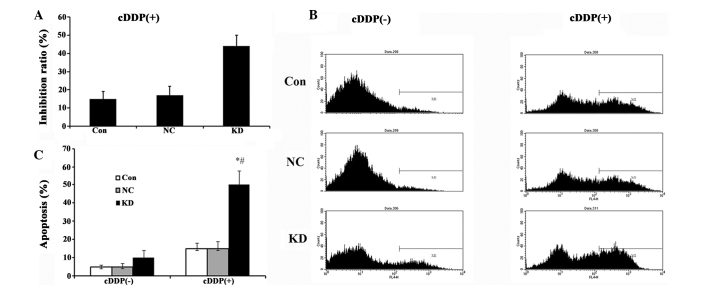
A549 cell proliferation and apoptosis in cells by transfection with lentivirus expressing KD RNAi, NC-RNAi or the parent A549 cells as CON combined with CDDP. (A) Cytotoxicity measured using a 3-(4,5-dimethylthiazol-2-yl)-2,5-diphenyltetrazolium bromide assay. Proliferation in A549 cells was significantly inhibited by ILK RNAi and CDDP. (B) Apoptosis measured by flow cytometry. (C) Statistical comparisons. The results demonstrated that transfection with KD and CDDP treatment significantly enhanced cell apoptosis. Data are presented as the mean ± standard deviation of a representative experiment performed in triplicate (n=3). ^*^P<0.05, compared with the Con group; ^#^P<0.05, compared with the NC group. NC, cells transfected with negative control RNAi; CON, control; KD, cells transfected with ILK specific RNAi; ILK, integrin-linked kinase; CDDP, cisplatin.

To assess the effects of ILK knockdown on cell proliferation of the cell line, A549, an MTT assay was performed. As expected, cells with ILK RNAi exhibited an inhibited cell proliferation ability compared with the control cells (Fig. 2A). In line with the MTT assay, the quantity and size of the colony in the ILK RNAi-infected cells were also significantly decreased compared with the control cells (Fig. 2B). In order to elucidate whether lentivirus-mediated ILK RNAi had any effects on the cell cycle of A549 cells, all three groups of A549 cells were subjected to a flow cytometry assay after 3 days of infection. The results revealed that ILK-shRNA-lentivirus infected cells exhibited an increased proportion in G0/G1 phase compared with the control-shRNA-lentivirus infected cells (Fig. 2C). As shown in Fig. 3B and C, although levels of cell apoptosis were higher in the ILK knockdown groups than that in the normal and control groups, no significant differences were observed, implying that downregulation of ILK alone is not an optimal approach for the treatment of lung cancer.

### Synergistic effects between CDDP and ILK RNAi on proliferation and apoptosis in A549 cells

Using drug sensitivity analysis, it was found that 10% CDDP treatment produced the maximal decrease in cell viability (5% CDDP, 0.876±0.015 vs 10% CDDP, 0.921±0.009, P<0.05; no difference between 10, 15, 20 and 25% treatment was observed, 0.921±0.009 vs 0.934±0.005, 0.934±0.004, 0.941±0.003; P>0.05). Therefore, proliferation and apoptosis in A549 cells undergoing ILK RNAi infection and 10% CDDP treatment was evaluated. Cells infected with ILK specific-RNAi and addition of CDDP exhibited significantly increased apoptotic levels and inhibited cell viability compared with the control cells (Fig. 3A, B and C), indicating a synergistic effect between CDDP and ILK RNAi.

### Effect of downstream gene expression of ILK on the regulation of cell survival and apoptosis

**Figure 4:**
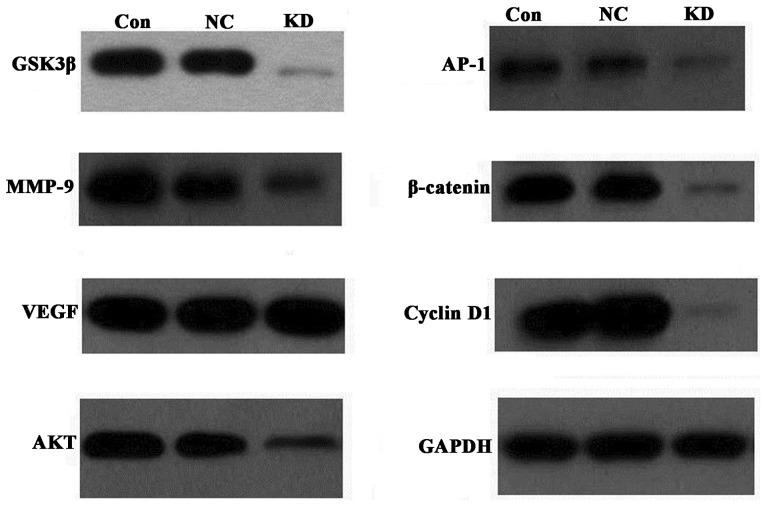
Effect of ILK knockdown on downstream protein expression by western blotting. The protein expression of GSK3β, AKT, AP-1, β-catenin, cyclin D1 and MMP-9 were found to be downregulated in the ILK specific-RNAi transfected cells. VEGF demonstrated no change following ILK knockdown. GAPDH was used as an internal control. CON, parent cells; NC, cells transfected with negative control RNAi; KD, cells transfected with ILK specific RNAi; GSK, glycogen-synthase kinase; VEGF, vascular endothelial growth factor; ILK, integrin-linked kinase; AP, activator protein; MMP, matrix metalloproteinase.

In order to investigate how cell survival and apoptosis were affected by ILK RNAi and CDDP, whether ILK RNAi regulated downstream gene expression was investigated. It was found that MMP-9, p-GSK3β, p-AKT, AP-1, β-catenin and cyclin D1 protein levels were downregulated in the ILK specific-RNAi trans-fected cells. VEGF exhibited no change following ILK knockdown (Fig. 4).

## Discussion

A significant number of studies have identified that ILK is a potential oncogene and inhibition of ILK by siRNA (15,16) or small molecules (18) may be a potentially useful therapeutic approach for treating cancer. However, whether the knockdown of ILK affects growth and apoptosis of lung cancer cells remains to be elucidated. In the present study, data revealed that lentivirus-mediated ILK gene silencing may significantly inhibit A549 cell proliferation and alter cell cycle progression. However, treatment with ILK RNAi alone had significant effects on cell apoptosis. CDDP-based combination chemotherapy is currently one of the most active treatments for advanced lung cancer. The mechanism of cisplatin-based chemotherapy is generally accepted as its ability to form adducts with DNA and cause DNA strand breaks in the nucleus, which interferes with normal transcription and/or DNA replication, leading to either repair of the DNA damage and cell survival or activation of the irreversible cell death program as a consequence (4,5). However, in the present study no significant differences in apoptosis in parent A549 cells and negative control-RNAi transfection cells with or without CDDP treatment were identified. This may be attributed to cisplatin insensitivity of A549 cell lines (19). In consideration of the mechanism of the above two approaches, lentivirus-mediated ILK siRNA and cisplatin-based chemotherapy were combined to investigate whether there is a synergistic interaction between them to promote cell apoptosis and inhibit cell growth. As expected, the present results demonstrated that apoptosis was signifi-cantly increased in cells infected with ILK specific-RNAi and addition of CDDP compared with other groups (Fig. 3). These findings suggest combined treatment modalities with lentivirus-mediated ILK interference and cisplatin chemotherapy may be more effective for cell apoptosis induction than mono-chemotherapy or knockdown. This contrasted with the conclusion drawn from the study by Kalra *et al* (20), who demonstrated that combination of CDDP and QLT0267, an ILK inhibitor, produced antagonistic interactions in a breast cancer model. This may result from the different pharmacological effects of these two compounds.

Furthermore, the present results also revealed that ILK siRNA may affect cell growth and apoptosis by regulating its downstream genes, including p-GSK3β, p-AKT, AP-1, β-catenin, cyclin D1 and MMP-9. Indirectly, it was also demonstrated that these downstream genes may mediate cisplatin resistance in lung cancer cells. These conclusions appeared to be in accordance with previous studies: ILK kinase activity is rapidly stimulated by the engagement of inte-grins to the extracellular matrix components. These stimuli result in activation of protein kinase B/Akt, suppression of apoptosis and promotion of cell survival. Thus, targeting inhibition of ILK led to low expression of p-Akt and promoted cell apoptosis (21,22). Additionally, Akt activity is reported to be a determinant of CDDP resistance (23–25). Therefore, reduced expression of p-Akt may reduce this resistance, further inducing cell apoptosis. In addition to regulating the activity of PKB/Akt, ILK also inhibits the activity of GSK-3 by phosphorylation at Ser9 (26). Downregulation of ILK led to a decrease in p-GSK3β and an increase in GSK-3 activity, which has been demonstrated to facilitate the cell apoptosis pathway (27–29). Further studies indicate that GSK-3 may be involved in cancer cell cycle arrest and apoptosis by regulating cyclin D1 expression, nuclear translocation of β-catenin and activation of the transcription factor AP-1 (26,30). Cyclin D1 is frequently overexpressed in lung cancer patients (31) and associated with poor survival of patients with lung cancer (32). Non-small cell lung cancer cells transfected with cyclin D1-targeted siRNA exhibited a marked decrease in cell growth rate and invasive capacity (33). AP-1 is a major transcription factor that regulates MMP-9 expression, which may contribute to the lower invasiveness and growth potential of cancer cells (34,35).

In conclusion, the results from the present study have identified for the first time, to the best of our knowledge, that downregulating ILK expression inhibits proliferation and cell cycle arrest in lung cancer cells. Downregulation of ILK expression and CDDP treatment, in combination, is a more effective approach for the treatment of lung cancer through affecting downstream gene expression, including p-GSK3β, p-AKT, AP-1, β-catenin, cyclin D1 and MMP-9.
